# *miR-17-5p*-Mediated RNA Activation Upregulates *KPNA2* Expression and Inhibits High-Glucose-Induced Apoptosis of Sheep Granulosa Cells

**DOI:** 10.3390/ijms26030943

**Published:** 2025-01-23

**Authors:** Yong Wang, Feng Tian, Sicong Yue, Jiuyue Li, Ao Li, Yang Liu, Jianyong Liang, Yuan Gao, Shuyuan Xue

**Affiliations:** 1 Inner Mongolia Academy of Agriculture and Animal Husbandry Sciences, Hohhot 010030, China; wangyongkeyan0322@126.com (Y.W.); tianfeng1309802@126.com (F.T.); lijy009@163.com (J.L.); 15661271930@163.com (Y.L.); liangjy0403@163.com (J.L.); may5june6@yeah.net (Y.G.); 2 College of Animal Science and Technology, Hebei Agricultural University, Baoding 071000, China; yueyueyuexiaojuan@163.com (S.Y.); 15032109803@163.com (A.L.)

**Keywords:** high glucose, granulosa cell, *miR-17-5p*, *KPNA2*, apoptosis

## Abstract

The glucose metabolism homeostasis in the follicular fluid microenvironment plays an important role in follicular maturation and ovulation, and excessively high or low glucose concentrations have adverse effects on the differentiation of follicular granulosa cells (GCs). However, a limited number of microRNAs (miRNA) have been reported to be involved in glucose-stimulated GCs differentiation. In this study, we characterized the miRNA expression profiles of sheep ovarian GCs cultured in high-glucose and optimal glucose concentrations and focused on a differentially expressed miRNA: *miR-17-5p*, which may be involved in regulating high-glucose-induced GC apoptosis by targeting *KPNA2*. We found that overexpression of *miR-17-5p* significantly promoted GCs proliferation and inhibited cell apoptosis, while downregulated the mRNA and protein expression of apoptosis-related makers (Bax, Caspase-3, Caspase-9, and Bcl-2). In contrast to the classical mechanism of miRNA silencing target gene expression, *miR-17-5p* overexpression significantly upregulated the expression of target gene *KPNA2*. A dual luciferase reporter gene assay verified the targeted binding relationship between *miR-17-5p* and *KPNA2* promoter. Meanwhile, overexpression of *KPNA2* further promoted the downregulation of apoptosis-related genes driven by *miR-17-5p* mimics. Knockdown of *KPNA2* blocked the inhibitory effect of *miR-17-5p* mimics on the expression of apoptosis-related genes. Our results demonstrated that *miR-17-5p* activated the *KPNA2* promoter region and upregulated *KPNA2* expression, thereby inhibiting GCs apoptosis under high glucose.

## 1. Introduction

Follicular development of ruminants is nutrition sensitive. Long-term production practice and research have found that reasonable increase of dietary energy can not only increase the glucose concentration in follicular fluid but can also promote follicular maturation and ovulation [1]. An excessive energy level can lead to abnormal glucose and lipid metabolism, ovarian dysfunction, and apoptosis of granulosa cells (GCs) [2,3,4]. Therefore, glucose metabolism homeostasis in the blood circulation and follicular fluid microenvironment plays an important role in regulating follicle maturation and ovulation, which directly affects the subsequent lambing number, reproductive efficiency, and breeding economic benefits of maternal animals.

Granulosa cells are the main site of glucose metabolism in the follicle, which can generate energy substrates such as lactate and pyruvate through the glycolytic pathway to provide energy for oocyte maturation and follicle development [5]. Our previous study showed that high glucose concentration (33.6 mM) induced apoptosis and inhibited steroidogenesis in GCs [6]. In both human and mouse in vivo studies, it has been confirmed that high concentrations of glucose induce GCs apoptosis, triggering follicular degeneration or atresia [7,8]. However, the molecular mechanism by which glucose regulates follicular development is very complex, involving precise spatiotemporal expression of multiple regulatory factors within germ cells. Exploring the key genes and regulatory mechanisms of high-glucose induced granulosa cell apoptosis is of great significance for understanding the dynamic process of follicle development in ruminants under nutrient regulation.

microRNA (miRNA) is a class of endogenous non-coding single-stranded RNA molecules with a length of 18–24 nt, which can regulate gene expression at the level of chromatin structure, RNA editing, RNA stability, transcription and translation, and participate in a variety of key biological and cellular processes [9,10]. It is known that the vast majority of miRNAs silence target gene expression by directing specific degradation of target mRNA sequences or inhibiting protein translation [11,12]. However, recent studies have found that some miRNAs can also activate gene expression and play a positive regulatory role by targeting the non-coding regions of gene promoters [13]. For example, *miR-744* and *miR-1186* upregulate the expression of *Cyclin* by activating the *Cyclin B1* promoter region and affecting the growth of mouse prostate cancer cells in vitro [14]. In female reproduction, *miR-551b-3p* binds to the *STAT3* promoter complementary sequence, recruits RNA polymerase II and *TWIST1* transcription factors, activates *STAT3* transcription, and upregulates *STAT3* expression in ovarian cancer cells [15]. Thus, miRNAs regulate gene expression in complex and diverse ways. The miRNA-17–92 gene cluster is one of the most widely studied miRNA gene clusters, including *miRNA-17*, *miRNA-18*, *miRNA19a*, *miRNA-19b*, *miRNA-20*, and *miRNA-92* [16]. It has functions such as regulating cell proliferation [17] and apoptosis [18] and plays an important role in glucose and lipid metabolism [19]. As a member of the miRNA-17–92 cluster, *miR-17-5p* has been reported to be involved in the regulation of porcine granulosa cell differentiation [20]. However, its function and mechanism in ovine follicular GC apoptosis are still unclear.

In this study, we first focused on *miR-17-5p*, a miRNA related to GCs apoptosis, and found that high concentrations of glucose significantly downregulated the expression of *miR-17-5p* and that *miR-17-5p* had the function of inhibiting GCs apoptosis. Mechanism studies have shown that *miR-17-5p* targets the *KPNA2* promoter region, upregulates *KPNA2* expression, and thus inhibits granulosa cell apoptosis. Our results provide reliable genetic resources and theoretical support for understanding the dynamic process of follicle development under glucose regulation.

## 2. Results

### 2.1. Systematic Functional Analysis of Differentially Expressed miRNAs

**Figure 1:**
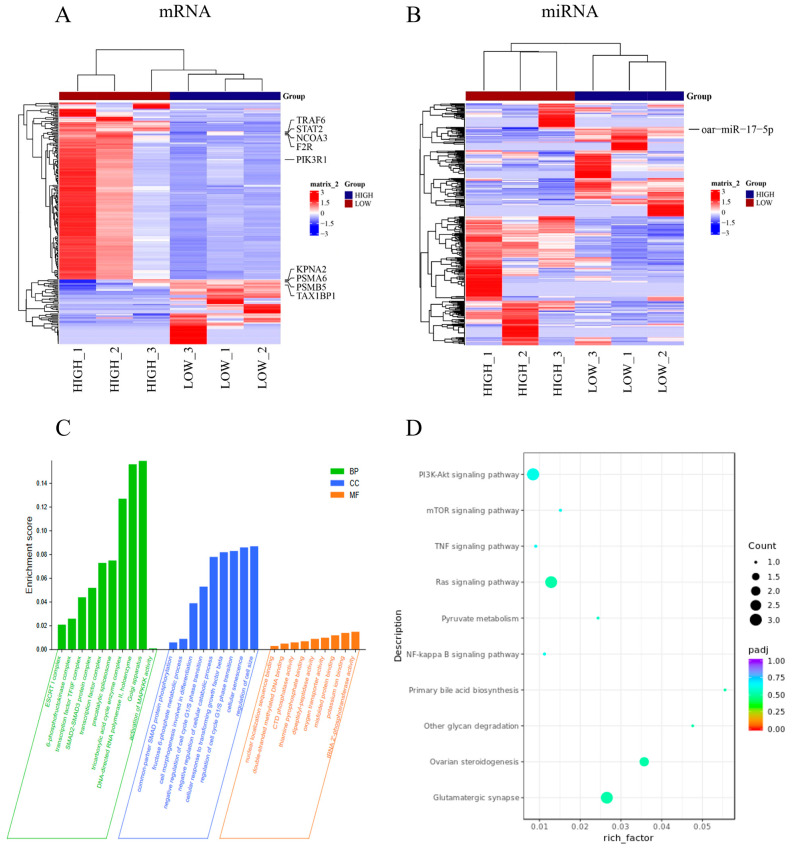
Systematic Functional Analysis of Differentially Expressed miRNAs. (**A**,**B**) Heat map of microRNA (miRNA) (**A**) and mRNAs (**B**) showing hierarchical clustering of changed miRNA and mRNAs of granulosa cells (GCs) in different glucose treatment groups; up and downregulated genes are colored in red and blue, respectively; (**C**) Gene Ontology (GO) categories of differentially expressed (DE) miRNA target genes in different glucose treatment groups; (**D**) Kyoto Encyclopedia of Genes and Genomes (KEGG) analysis of DE miRNA target genes in different glucose treatment groups. The size and color of each bubble represents the number of genes in each pathway and *p* value, respectively.

The GC samples were divided into Low and High groups for Small RNA sequencing (sRNA-seq), and the raw data quality situation showed that the GC content was higher than 46% and the Q30 was higher than 98% (Table S1). There were 796 (417 upregulated and 379 downregulated) DE mRNAs and 94 (47 upregulated and 47 downregulated) DE miRNAs identied in the high and low glucose-induced GCs groups (Figures S1 and S2). Hierarchical clustering of DE mRNAs (Figure 1A) and DE miRNAs (Figure 1B) revealed the expression patterns of the individuals in the high and low group comparisons. To study the function of observed changes in DE miRNAs in the high and low comparison groups, a Gene Ontology (GO) term enrichment analysis of the predicted target genes of DE miRNAs was performed. As shown in Figure 1C, the activation of MAPKKK activity, regulation of cell size, and tRNA 2-phosphotransferase activity were significantly enriched in the two groups, suggesting that concentrations of glucose may regulate GC function by regulating cell proliferation, apoptosis, and differentiation-related signaling pathway activity, as well as participating in RNA transcription and translation. Subsequently, Kyoto Encyclopedia of Genes and Genomes (KEGG) enrichment analysis in miRNA target genes was performed. The KEGG analysis revealed several significantly enriched pathways (Figure 1D), including PI3K-AKT signaling pathway, Ras signaling pathway, TNF signaling pathway, and NF-Kappa B signaling pathway. This indicates that DE miRNAs are involved in the regulation of granulosa cell apoptosis and growth by glucose.

### 2.2. miR-17-5p-Target Genes-Signaling Pathway Interaction Network

**Figure 2:**
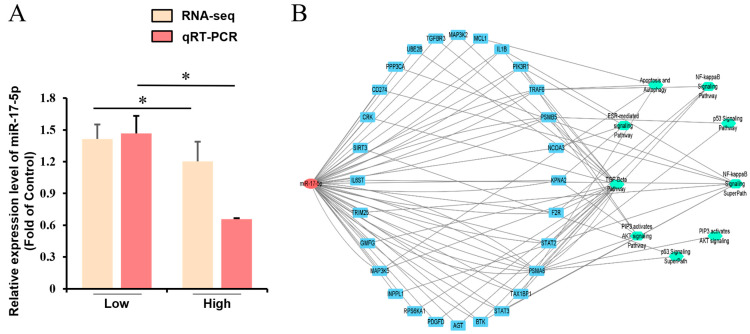
Prediction of the regulatory network of *miR-17-5p* in GCs. (**A**) Analysis of *miR-17-5p* expression in GCs cultured with different concentrations of glucose; (**B**) The *miR-17-5p*-target genes-signaling pathway interaction network. Values represent means ± SEM for three individuals. *, *p* < 0.05.

Based on the functional enrichment analysis of DE miRNAs, we hypothesized that DE miRNAs may affect granulosa cell apoptosis by regulating apoptosis-related signaling pathways (Table S2). We focused on one differential miRNA: *miR-17-5p*. The expression of *miR-17-5p* was significantly downregulated in the high-glucose group compared with the low-glucose concentration group, and the analysis of quantitative Real-time PCR (qRT-PCR) also verified this result (Figure 2A). Therefore, an *miR-17-5p*-target genes-signaling pathway interaction network was constructed in GCs, including 28 target genes and eight pathways (Figure 2B, Table S3).

### 2.3. The miR-17-5p Promotes Proliferation and Suppresses Apoptosis of Granulosa Cells (GCs)

**Figure 3:**
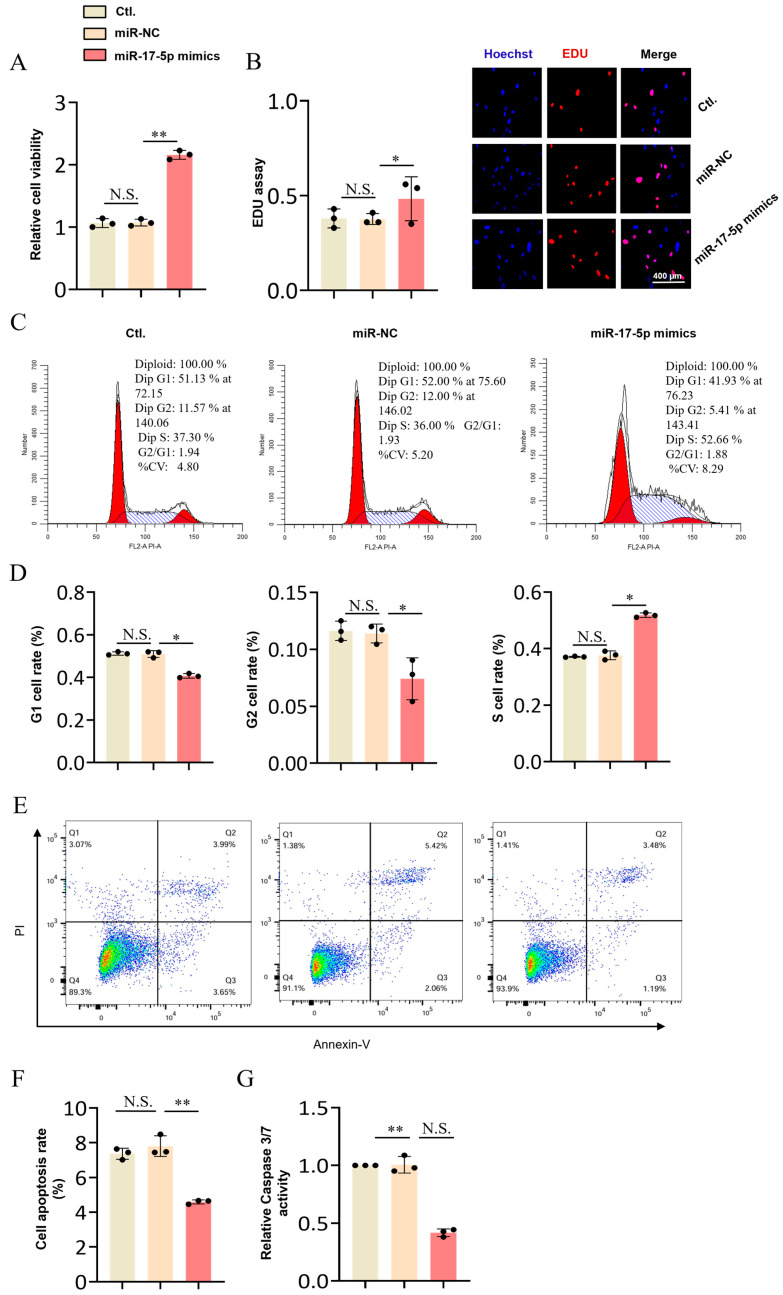
*miR-17-5p* promoted GCs proliferation and inhibited apoptosis. (**A**) The CCK-8 assay was performed to assess the effect of *miR-17-5p* mimics on GCs viability; (**B**) The EDU was used to detect the effect of *miR-17-5p* mimics on the proliferation of granulosa cells; (**C**,**D**) The EDU was used to detect the effect of *miR-17-5p* mimics on the proliferation of granulosa cells. Cell cycle analysis of GCs at 48 h after transfection of *miR-17-5p* mimics plasmid, cale bars are 200 µm; (**E**,**F**) The results of cell apoptosis were detected by flow cytometry after transfection with *miR-17-5p* mimic. The X-axis and Y-axis represent PI fluorescence and Annexin-V fluorescence. Q1, Q2, Q3, and Q4 represent dead cells, late withered, early withered, and live cells, respectively; (**G**) Caspase3/7 activity assay after transfection of *miR-17-5p* mimics in GCs. Values represent means ± SEM for three individuals. * *p* < 0.05; ** *p* < 0.01. N.S., not significant.

To examine the potential function of *miR-17-5p* in GCs apoptosis, we detected the proliferation and apoptosis of cells after *miR-17-5p* transfection. The CCK-8 and EDU staining showed that the proliferation rate of GCs transfected with *miR-17-5p* mimics was significantly increased compared with GCs transfected with miR-NC (Figure 3A,B). We found that the proportion of GCs cells entering G1 and G2 phase of the cell cycle decreased and the proportion of cells entering S phase increased after *miR-17-5p* transfection (Figure 3C,D). These results reveal that *miR-17-5p* could promote GCs proliferation. Meanwhile, the cell apoptosis assay showed that overexpression of *miR-17-5p* significantly reduced the GC apoptosis rate (Figure 3E,F) and caspase3/7 activity (Figure 3G) compared with that of the GCs transfected with miR-NC.

### 2.4. The miR-17-5p Regulates the Expression of Apoptosis-Related Genes and KPNA2

**Figure 4:**
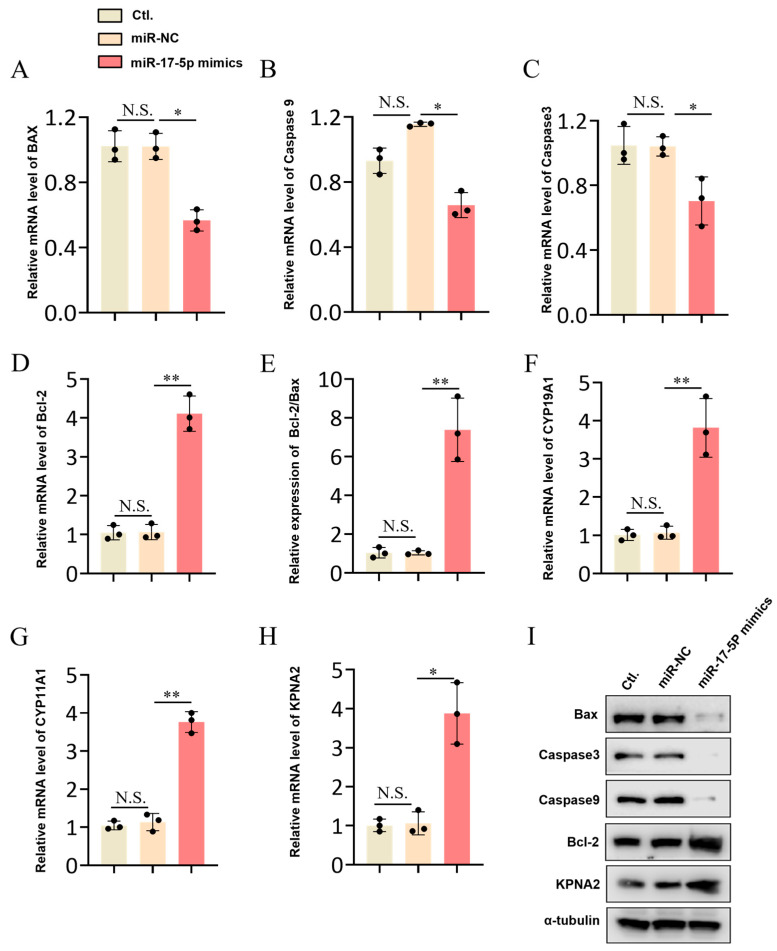
*miR-17-5p* affected the expression of apoptosis-related genes and *KPNA2*. (**A**–**H**) The quantitative Real-time PCR (qRT-PCR) of relative expression levels of apoptosis-related mRNAs (*Caspase-3*, *Caspase-9*, *BAX*, and *Bcl-2*), steroid synthesis-related mRNA (*CYP11A1* and *CYP19A1*) and target gene (*KPNA2*) in GCs transfected with miR-17-5p mimics. (**I**) Overexpression of *miR-17-5p* affected the expression of apoptosis-related proteins and KPNA2 proteins. Values represent means ± SEM for three individuals. * *p* < 0.05; ** *p* < 0.01. N.S., not significant.

We found overexpression of *miR-17-5p* significantly downregulated the expression of pro-apoptotic-related genes (*caspase-3*, *caspase-9*, and *bax*), and significantly upregulated the expression of anti-apoptotic gene *bcl-2* (Figure 4A–D). Consistently, the bcl-2/bax ratio was significantly higher in the *miR-17-5p* group than in the miR-NC group (Figure 4E). Furthermore, our study demonstrated that *miR-17-5p* significantly upregulated the expression of genes associated with steroid hormone synthesis, specifically *CYP11A1* and *CYP19A1* (Figure 4F,G). We predicted that Karyopherin-alpha2 (*KPNA2*) was a target gene of *miR-17-5p*, and further validation found that overexpression of *miR-17-5p* significantly promoted the expression of *KPNA2* (Figure 4H). The Western blot assay also showed that overexpression of *miR-17-5p* decreased the protein levels of bax, caspase-3, and caspase-9 but increased the protein levels of bcl-2 and KPNA2 (Figure 4I).

### 2.5. Binding of miRNA and KPNA2 Promoter Region Induces KPNA2 Transcriptional Activation

**Figure 5:**
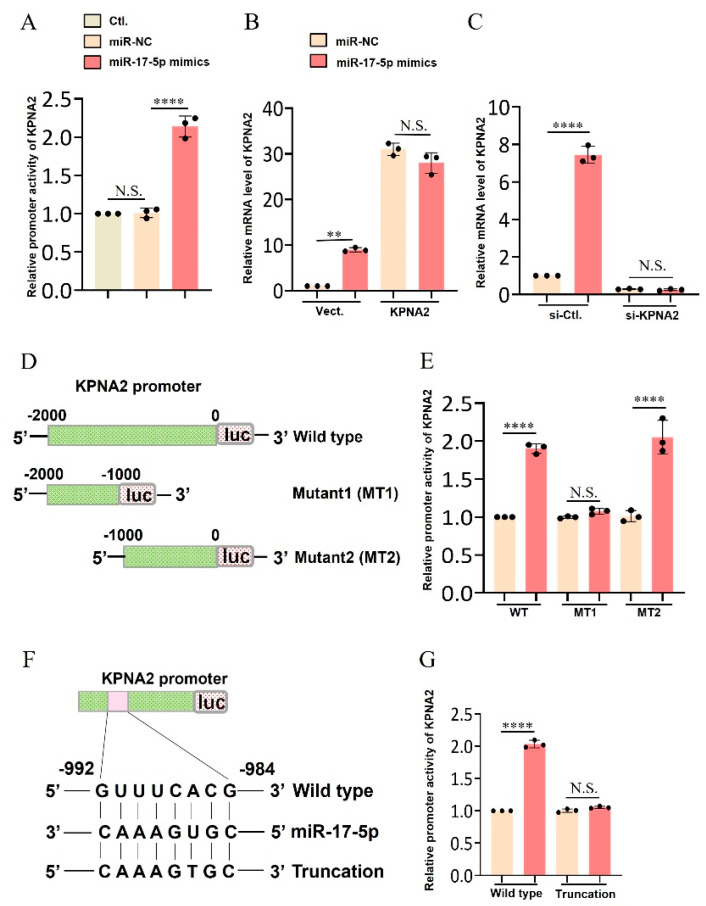
Validation of the binding relationship between *miR-17-5p* and *KPNA2* promoter. (**A**) Effect of overexpression of *miR-17-5p* on *KPNA2* mRNA expression. (**B**) *miR-17-5p* mimics and pcDNA3.1-*KPNA2* were co-transfected into GCs for qRT-PCR validations; (**C**) Western blot was used to determine the protein expression level of *KPNA2* after co-transfection of *miR-17-5p* mimics and specific siRNA of *KPNA2*; (**D**) The GCs were transfected with *miR-17-5p* mimics, vectors containing wild-type (KPNA2 WT), mutated *KPNA2* (Mut-1), or mutated *KPNA2* (Mut-2) followed by dual luciferase assay; (**E**) Dual luciferase assay was used to analyze the transcriptional activation of *KPNA2* promoter by *miR-17-5p*; (**F**) The truncated plasmid of *KPNA2* promoter region was constructed, and *miR-17-5p* and *KPNA2* truncated luciferase plasmid were co-transfected into sheep ovarian granulosa cells; (**G**) Dual luciferase assay was used to analyze the specific binding site of *miR-17-5p* to the *KPNA2* promoter region. Values represent means ± SEM for three individuals. ** *p* < 0.01; **** *p* < 0.0001; N.S., not significant.

Based on the above *miR-17-5p*–*KPNA2*-pathway network, several genes (e.g., *KPNA2*, *PIK3R1*, and *STAT3*) were predicted to be *miR-17-5p* target genes (Figure 2B). Interestingly/significantly, RNA-seq analysis showed that the expression of both *miR-17-5p* and its target gene *KPNA2* was significantly downregulated under high glucose concentration (Figure 1A,B), and overexpression of *miR-17-5p* significantly promoted the mRNA expression of *KPNA2* (Figure 5A). In addition, co-transfection of *miR-17-5p* mimics plasmid and *KPNA2* overexpression plasmid (pcDNA3.1–*KPNA2*) in GCs showed that *KPNA2* overexpression further upregulated the *miR-17-5p*-induced *KPNA2* expression (Figure 5B). Conversely, we added *KPNA2* inhibitor to the *miR-17-5p* mimic group and found that *KPNA2* inhibitor can rescue *miR-17-5p*-induced upregulation of the *KPNA2* expression level (Figure 5C). These results implied that *miR-17-5p* may directly target *KPNA2* and promote *KPNA2* expression.

To further investigate the binding relationship between *miR-17-5p* and *KPNA2*, dual luciferase reporter constructs containing miRNA response elements (MRE; Wild-type (WT)), mutant 1 (MT1) plasmid, and mutant (MT2) plasmid were co-transfected into GCs with *miR-17-5p* mimics (Figure 4G). These results indicated that *miR-17-5p* had a direct binding relationship with *KPNA2*, and the targeted binding site of *miR-17-5p* was within 1000 bp of the 3′ end of *KPNA2*. On the basis of these results, we constructed an order short plasmid in the *KPNA2* promoter region by software prediction and further analyzed the specific binding site of *miR-17-5p* to the *KPNA2* promoter region by dual luciferase reporter assay. The sequence “GUUUCACG” (5→3′) was demonstrated as *miR-17-5p*-targeted motif in the *KPNA2* gene.

### 2.6. The miR-17-5p/KPNA2 Pathway Regulates the Expression of Apoptosis-Related Genes, Promotes Granulosa Cell Activity, and Inhibits Cell Apoptosis

**Figure 6:**
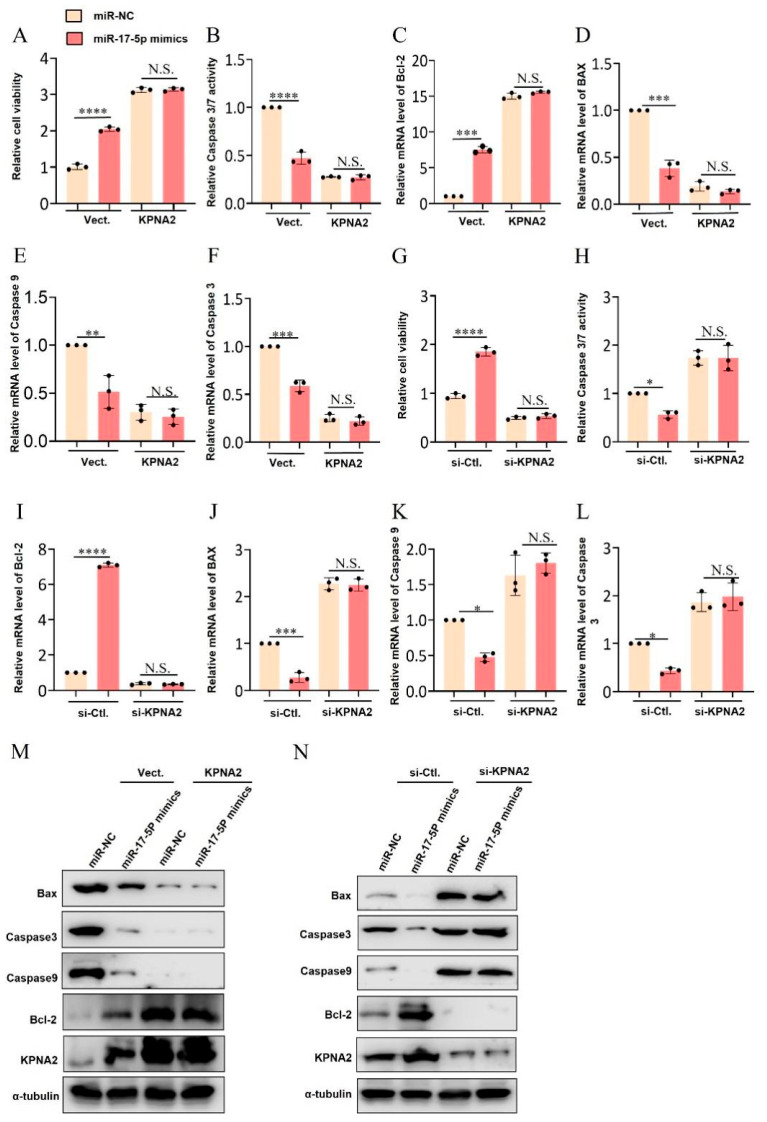
*miR-17-5p*-dependent *KPNA2* regulates granulosa cell proliferation and apoptosis. Validation of the binding relationship between *miR-17-5p* and *KPNA2* promoter. (**A**–**F**) After co-transfection of *miR-17-5p* mimics and *KPNA2* overexpression vector, the proliferation, apoptosis, and apoptosis-related gene expression of GCs were measured; (**G**–**L**) After co transfection of *miR-17-5p* mimics and *KPNA2* small interfering RNA, the proliferation, apoptosis, and expression of apoptosis-related genes in granulosa cells were detected; (**M**) The effect of co-transfection of *miR-17-5p* mimics and *KPNA2* overexpression plasmids on the expression of apoptosis-related proteins in GCs; (**N**) The effect of co-transfection of *miR-17-5p* mimics and *KPNA2* small interfering RNA on the expression of apoptosis-related proteins in GCs. Values represent means ± SEM for three individuals. * *p* < 0.05; ** *p* < 0.01; *** *p* < 0.001; **** *p* < 0.0001. N.S., not significant.

To further explore whether *miR-17-5p* inhibits cell apoptosis via promoting *KPNA2* expression, we co-transfected *miR-17-5p* mimics plasmid and *KPNA2* overexpression vector (pcDNA3.1–*KPNA2*) into GCs. We confirmed that co-transfection of *miR-17-5p* mimics and *KPNA2* overexpression plasmid significantly increased cell viability and reduced caspase3/7 activity compared with that of the *miR-17-5p* mimics transfected cells (Figure 6A,B). Meanwhile, we found *KPNA2* overexpression further promoted the *miR-17-5p*-induced upregulation of anti-apoptosis-related mRNA (*bcl-2*) expression and downregulation of apoptosis-related mRNAs (*bax*, *caspase-9* and *caspase-3*) expression (Figure 6C–F). This result was also verified at the protein level by western blot assay (Figure 6M).

Conversely, we co-transfected *miR-17-5p* and *KPNA2* small interfering RNA (si-*KPNA2*) into GCs. Results showed that *KPNA2* knockdown significantly reversed the increased proliferation of GCs expression of senescence markers, as revealed by CCK-8 analysis, and mitigated the decreased cellular apoptosis induced by *miR-17-5p* overexpression, as indicated by caspase3/7 Activity assay (Figure 6G,H). The qRT–PCR and western blot indicated decreased apoptosis-related genes and proteins expression in the *miR-17-5p* mimics group, which enhanced adding si-*KPNA2* treatment, while the si-*KPNA2* treatment significantly inhibited anti-apoptosis-related genes upregulation by *miR-17-5p* mimics (Figure 6I–L,N).

## 3. Discussion

Glucose, as the most basic energy supply substance in biology, is the primary source of cell metabolism and body energy. In recent years, studies in female reproduction have found that the occurrence and development of endometriosis [21], polycystic ovary syndrome [22], and other diseases are often accompanied by abnormal glucose metabolism in ovary GCs. With the development of bioinformatics and experimental technology, more and more miRNAs have been confirmed to play important functions in reproduction. In this study, we identified a novel inhibitor of GC apoptosis—*miR-17-5p*—and found that *miR-17-5p* was involved in the regulation of cell apoptosis under high glucose. In other cell types such as brain endothelial cells [23], thyroid cancer cells [24], and hepatocellular carcinoma cells [25], *miR-17-5p* has been shown to function as an apoptotic modulator. This study presents the identification of a potential small molecule that can mitigate fertility decline in females caused by glucose metabolism disorders. This discovery enhances our understanding of the mechanisms underlying nutrient regulation during the dynamic development of ruminant follicles.

With the occurrence of follicular waves, mammalian ovarian and follicular development is a cyclical and dynamic process [26]. Given the dynamic nature of mammalian follicle development, miRNAs have been predicted to play important regulatory roles in multiple aspects, such as follicle function [27] and corpus luteal development [28], as well as ovarian diseases [29]. Studies on mouse cumulus cells have found that miRNAs play a critical role in glucose metabolism of ovarian GCs, with *miR-23b-3p*, *let-7b-5p*, *34b-5p*, and *145a-5p* involved in the regulation of glycolysis, while *miR-24-3p*, *3078-3p*, *183-5p*, and *7001-5p* inhibit the pentose phosphate pathway of CCs [30]. Our previous study found that high concentrations of glucose (33.6) could inhibit GCs proliferation, glycolytic metabolism, and steroid hormone production, and 8.4 mM glucose represents an optimum concentration for glycolysis and steroid hormone secretion [6]. In the present study, we discovered a new signaling pathway, the *miR-17-5p*/*KPNA2* pathway, that contributes to high glucose-induced GCs apoptosis and enriches the molecular function of *miR-17-5p* in GCs. KPNA2, as an adaptor protein for nuclear transport, has been shown to be involved in the regulation of reproduction [31]. *KPNA2* deficiency results in defective zygotic genome activation and arrested embryo development [32]. Therefore, identification of the functional characteristics of *miR-17-5p* during follicular development can provide a valuable reference for in-depth understanding of the dynamic development process of ruminant follicles.

Bioinformatics analysis software can predict the binding sites between miRNA and mRNA through specific computational algorithms [33]. Therefore, the important functions and mechanisms of miRNA during follicular development have been further revealed. Most studies in ovine follicles have shown that miRNAs silence post-transcriptional gene expression by targeting the 3′-untranslated and/or coding regions of mRNA or inhibit protein translation of target genes by binding to the 5′-untranslated regions of mRNA. For example, *miR-346* is involved in regulating the proliferation of Husheep ovarian GCs by targeting the *LIF*/*STAT3* signaling pathway [34], while *miR-27a-3p* inhibits *CYP19A1* expression in granulosa cells, thereby inhibiting estrogen synthesis [35]. Recent studies have found that miRNA can also play a positive regulatory role by activating the non-coding region of the promoter to induce the expression of target genes [13]. This regulatory mode of inducing gene-transcription activation (RNAa) was first reported in human cell lines. Researchers designed and synthesized Small Double-stranded RNAs (dsRNAs) targeting 21 nt of human gene promoters. The dsRNAs were found to activate the expression of target genes specifically and permanently [36]. Similarly, our study first discovered that *miR-17-5p* binds to the *KPNA2* promoter region in sheep GCs, inducing *KPNA2* transcriptional activation, promoting GCs proliferation, and inhibiting apoptosis. The non-classical regulatory mechanism of miRNA-mediated RNA activation promoting gene expression is enriched in sheep germ cells, which provides new clues for the study of miRNA in ruminants.

## 4. Materials and Methods

### 4.1. Isolation and Culture of GCs

Details of granulosa cells collection, isolation, and culture have been described previously [6]. Briefly, ewe ovaries were removed from Thin-tailed Han sheep (ages ranged from 1 to 1.5 years) at a local slaughterhouse (Tang County, Baoding, China) and returned to the laboratory within 2 h in buffered saline solution at 37 °C. Ovaries were rinsed at least three times with saline and 1× PBS, respectively. To eliminate any individual effects, follicular fluid was collected from at least 60 follicles 1–3 mm in diameter using a 20 mL disposable syringe. GCs were harvested after the pooled follicle suspensions were centrifuged at 1000× *g* for 10 min. Then, the GCs were seeded in cell culture plates (ThermoFisher Scientific, Waltham, MA, USA) at a density of 2 × 10^5^/well. Granulosa cells were cultured in DMEM F12 medium (Gibco, Waltham, MA, USA) supplemented with 10% fetal bovine serum and 1% streptomycin/penicillin mixture, and the cells were placed in a humidified atmosphere of 37 °C and 5% CO_2_ for 48 h.

The original medium was removed when the cells reached 70% of the medium. Cells were cultured in DMEM without serum, pyruvate, glucose, or phenol red (Solarbio, Beijing, China) for 8 h. The GCs were divided into three groups with three replicates per group and cultured with glucose at concentrations of 8.4 mM and 33.6 mM. The 8.4 mM group was the low-concentration group (Low), representing the optimal glucose concentration for proliferation and differentiation of ewe GCs in vitro [6]. The 33.6 mM concentration group was the high-concentration group (High), representing a high glucose concentration that far exceeds the physiological concentration of follicle [6,37,38]. Two groups of granulosa cells were harvested after 24 h and used for subsequent RNA sequencing.

### 4.2. RNA Extraction, Library Construction, and Small RNA Sequencing

The sRNA sequencing was performed on GCs in the low-glucose and high-glucose groups. Total RNA of the two groups were isolated using TRIzol Reagent (Invitrogen, Carlsbad, CA, USA), and the purity (OD 260/280) and concentration of extracted RNA were evaluated by Nanodrop 2000 [39]. For miRNA, total RNA was used as the starting sample, and the two ends of sRNA were directly connected with the linker, and then synthesis of complementary DNA (cDNA) by reverse transcription. To obtain cDNA library after PCR amplification, the target DNA fragments were separated by PAGE gel electrophoresis. According to the requirement of specified concentration and target data volume, different libraries were combined and sequenced by HiSeq (Illumina HiSeqTM2500, San Diego, CA, USA).

### 4.3. Data Processing and Differential miRNAs Expression Identification

To ensure the quality of sequencing data, raw data quality must be evaluated, filtered, and screened. The GC content, Q20, and Q30 percentages of clean data were evaluated, and the clean reads of each sample were screened for sRNAs within a certain length range for subsequent analysis. The distribution of sRNAs on the reference sequence was analyzed by positioning the length-screened sRNAs to the reference sequence using Bowtie (Irvine, CA, USA) [40,41].

The expression amount of known and novel miRNAs in each sample was counted and Transcripts Per Kilobase of exon model per Million mapped reads (TPM) conversion was performed to obtain the expression level of the transcript. The data of differentially expressed miRNAs were analyzed using DESeq2 (version 1.34.0) based on negative binomial distribution [42]. Differential miRNAs were screened from two aspects: fold change and corrected significance level (*p*-adj/q-value).

### 4.4. Target Gene Prediction and Functional Annotation Analysis

The miRanda 3.3a (http://www.microrna.org, accessed on 12 July 2024) [43], miRDB (https://mirdb.org/, accessed on 12 July 2024) and TargetScan (http://www.targetscan.org/, accessed on 20 July 2024) software packages [44] were adopted to predict potential target mRNAs. Gene Ontology (GO, http://www.geneontology.org/) and Kyoto Encyclopedia of Genes and Genomes (KEGG, http://www.genome.jp/kegg/, accessed on 26 July 2024) databases were used to annotate candidate target genes to better understand the function of target genes of DE miRNAs and their corresponding metabolic networks.

### 4.5. Plasmid Construction, RNA Oligonucleotides, and Cell Transfection

The *KPNA2* overexpression construct was generated by amplifying the *KPNA2* coding sequence, which was subsequently integrated into the HindIII/KpnI restriction sites of the pcDNA3.1 overexpression plasmid (named pcDNA3.1-*KPNA2*).

The siRNA target against the *KPNA2* gene (si-*KPNA2*), siRNA nonspecific vector (vect.), non-specific control (ctl.), mimic negative control (miR-NC), and *miR-17-5p* mimics were synthesized by RiboBio (Guangzhou, China).

*miR-17-5p* binding sites in *KPNA2* 3′UTR was amplified by PCR using a cDNA template synthesized from total RNA. Then, the PCR products were subcloned into XhoI/XbaI restriction sites in the pmirGLO dual-luciferase reporter vector to generate the pmirGLO-Luc-*KPNA2* reporter.

The GCs were seeded in 96-well (5 × 10^3^ cells/well), 12-well (1 × 10^5^ cells/well), or six-well (1 × 10^6^ cells/well) cell culture plates until the cell density reached 60–70% (RiboBio, Guangzhou, China). All transfections were detected using Lipofectamine TM 2000 (Invitrogen, Carlsbad, CA, USA), and synthetic liposomes were performed according to the manufacturer’s directions. Cells from each group were collected 24 h after transfection. The oligonucleotide sequences information used in study were shown in Supplementary Table S4.

### 4.6. Cell Proliferation Assay

After transfection, GCs proliferation was detected using the Cell Counting Kit-8 (TransGen Biotech, Beijing, China) by adding 10 µL of CCK-8 solution to each well and incubating for 1–4 h in a cell incubator. Subsequently, the absorbance at a wavelength of 450 nm was determined using a microplate reader (BioTek, Shoreline, WA, USA).

### 4.7. 5-Ethynyl-2′-deoxyuridine (EdU) Assay

Cells were seeded into 12-well plates and then transfected with miRNA mimics. After that, the proliferation assay (EDU, Beyotime, Shanghai, China) was added to the GCs following the manufacturer’s protocol. Finally, images were randomly captured and collected using fluorescence microscopy (DMi8; Leica, Wetzlar, Germany).

### 4.8. Cell Cycle Assay

After transfection, the cells were fixed with a final concentration of 70% ethanol for more than 4 h. The fixed cells were incubated with 100 µL of RNase in the dark at 37 °C for 30 min. The cells were then labelled with 400 µL of propidium iodide (PI) for 30 min and processed in flow cytometer (BD Biosciences, San Jose, CA, USA).

### 4.9. Cell Apoptosis Analysis

After transfection for 24 h, the GCs were digested into centrifuge tubes. The cells were stained with FITC Annexin V (AnV) (FITC Annexin V Apoptosis Detection Kit I, BD Biosciences, NJ, USA) and propidium iodide (PI), the data of cell apoptosis was calculated using FlowJo software (version 7.6.5).

### 4.10. Caspase3/7 Activity Assay

Primary GCs were seeded in 96-well plates. After transfection for 24 h, GCs cells were subjected to the caspase3/7 activity assay by Caspase-Glo_3/7 Assay Systems (Promega, #G8091, Madison, WI, USA) according to the manufacturer’s instructions. The assay was conducted in triplicate and repeated independently three times, which was represented as a fold increase in fluorescence calculated by comparing cells with untreated control cells.

### 4.11. RNA Extraction, Complementary DNA (cDNA) Synthesis, and RT-qPCR

After transfection, total RNA was extracted from cultured cells using TRIzol reagent (Invitrogen, Life Technologies, Carlsbad, CA, USA) according to the manufacturer’s instructions. The OD260, OD280, and OD260/OD280 values were determined by microspectrophotometer, and the purity and concentration of the RNA were calculated. The cDNA synthesis of the mRNAs was carried out using the PrimeScript RT Reagent Kit With gDNA Eraser (Takala, Beijing, China), and the ReverTra Ace qPCR RT Kit (Toyobo, Osaka, Japan) was used for synthesizing the cDNA of miRNA. The RT–qPCR analysis of mRNAs and miRNA were performed in an Applied Biosystem^®^StepOnePlus™ system (Thermo Fisher Scientific Inc., Carlsbad, CA, USA). Primers of miRNA and mRNAs were designed using RiboBio (RiboBio, Guangzhou, China), and U6 and GAPDH were regarded as endogenous controls for miRNA and mRNAs, respectively. Information of primers was listed in Table S5. Data analyses were performed using the 2^−ΔΔCt^ method.

### 4.12. Western Blotting

Total proteins from tissues and cells were lysed conforming to the user’s guidebook of RIPA lysis buffer (Beyotime, Shanghai, China); this was followed by separation with 10% sodium dodecyl sulfate–polyacrylamide gel electrophoresis (SDS–PAGE) and transfer with polyvinylidene difluoride (PVDF) membranes (Bio-Rad, Hercules, CA, USA). After incubation with the indicated primary and secondary antibodies, signals were visualized by ECL. Membrane was then ready for scanning by Image studio system. Protein quantification was conducted by ImageJ software (version 1.8.0). The antibody used included the Bcl-2, Bax, Caspase-3, Caspase-9, KPNA2, anti-Argonaute-2, and anti-β-actin were purchased from abcam group (Cambridge, UK). The goat anti-rabbit IgG (H + L)-HRP (1:5000; catalogue no. ab6721, Abcam, Cambridge, UK) was used as a secondary antibody.

### 4.13. Dual-Luciferase Reporter Assay

Approximately 3 × 10^4^ GCs were plated onto 24-well tissue culture plates 24 h before transfection. Cells were transfected with a mixture of Renilla luciferase and indicated luciferase reporters using Lipofectamine 2000 (Invitrogen, Carlsbad, CA, USA). Forty-eight hours after transfection, the cells were harvested and subjected to an assay by using the Dual Luciferase Reporter Assay system (Promega, Madison, WI, USA). The luciferase activity was detected by the Fluorescence/MultiDetection Microplate Reader (BioTek, Winooski, VT, USA). The relative luciferase activities were normalized with the Renilla luciferase activities.

### 4.14. Statistical Analysis

Each experiment was performed at least three times. All data were normally distributed continuous variables and reported as the mean ± standard error of the mean (SEM). The statistics of all trials except sequencing data were analyzed by SPSS version 22.0 (SPSS Inc., Chicago, IL, USA). One-way analysis of variance (ANOVA) and Tukey’s test were used to assess statistical significance. A *p* < 0.05 was considered statistically significant.

## 5. Conclusions

In summary, we evaluated the miRNA expression profile of sheep ovarian GCs treated with different doses of glucose and found that *miR-17-5p* is involved in the regulation of high-glucose-induced GCs apoptosis. In addition, it was further found that *miR-17-5p* promoted GCs proliferation and inhibited apoptosis by upregulating the expression of its target gene *KPNA2*, which provided reliable genetic resources and theoretical support for understanding the dynamic process of follicle development under the regulation of nutrients.

## Data Availability

The data sets used during the current study are available from the corresponding author on reasonable request.

## Supplementary Materials

The following supporting information can be downloaded at: https://www.mdpi.com/article/10.3390/ijms26030943/s1.

## References

[1] Guo Y.X., Duan C.H., Hao Q.H., Liu Y.Q., Li T., Zhang Y.J. (2019). Effect of short-term nutritional supplementation on hormone concentrations in ovarian follicular fluid and steroid regulating gene mRNA abundances in granulosa cells of ewes. Anim. Reprod. Sci..

[2] Nascimbeni F., Salda A.D., Carubbi F. (2018). Energy balance, glucose and lipid metabolism, cardiovascular risk and liver disease burden in adult patients with type 1 Gaucher disease. Blood Cells Mol. Dis..

[3] Gao Y.Y., Zou Y.G., Wu G.J., Zheng L.W. (2023). Oxidative stress and mitochondrial dysfunction of granulosa cells in polycystic ovarian syndrome. Front. Med..

[4] Wang Y., Guo Y.X., Duan C.H., Yang R.C., Zhang L.C., Liu Y.Q., Zhang Y.J. (2022). Long Non-Coding RNA GDAR Regulates Ovine Granulosa Cells Apoptosis by Affecting the Expression of Apoptosis-Related Genes. Int. J. Mol. Sci..

[5] Zhang X.D., Zhang W.B., Wang Z.J., Zheng N.N., Yuan F.F., Li B., Li X.L., Deng L., Lin M., Chen X. (2022). Enhanced glycolysis in granulosa cells promotes the activation of primordial follicles through mTOR signaling. Cell Death Dis..

[6] Wang Y., Duan C.H., Guo Y.X., Li J.J., He H.Y., Li R.T., Zhang Y.J., Liu Y.Q. (2022). Effects of glucose on glycolysis and steroidogenesis as well as related gene expression in ovine granulosa cells in vitro. Small Rumin. Res..

[7] Zhang N., Shan Y.H., Lian H.Y., Ma X.H., Zhang W., Liu X.Y., Zhuang L.L., Liu X.N., Liu Y., Zheng K.X. (2023). Resveratrol protects against high glucose-induced steroidogenesis and apoptosis in murine granulosa cells. Cell Mol. Biol..

[8] Samie K.A., Tabandeh M.R., Afrough M. (2020). Betaine ameliorates impaired steroidogenesis and apoptosis in mice granulosa cells induced by high glucose concentration. Syst. Biol. Reprod. Med..

[9] Bhattacharya A., Jha V., Singhal K., Fatima M., Singh D., Chaturvedi G., Dholakia D., Kutum R., Pandey R., Bakken T.E. (2021). Multiple Alu Exonization in 3’UTR of a Primate-Specific Isoform of CYP20A1 Creates a Potential miRNA Sponge. Genome Biol. Evol..

[10] Francis M., Grider A. (2018). MiRNA-target interactions in osteogenic signaling pathways involving zinc via the metal regulatory element. BioMetals.

[11] Xu G.C., Meng Y., Wang L.H., Dong B., Peng F.F., Liu S.T., Li S.K., Liu T. (2021). MiRNA-214-5p inhibits prostate cancer cell proliferation by targeting SOX4. World J. Surg. Oncol..

[12] Liu J.H., Liang Y., Qiao L.Y., Xia D., Pan Y.Y., Liu W.Z. (2022). MiR-128-1-5p regulates differentiation of ovine stromal vascular fraction by targeting the KLF11 5′-UTR. Domest. Anim. Endocrinol..

[13] Vaschetto L.M. (2018). miRNA activation is an endogenous gene expression pathway. RNA Biol..

[14] Huang V., Place R.F., Portnoy V., Wang J., Qi Z.X., Jia Z.J., Yu A., Shuman M., Yu J.W., Li L.C. (2012). Upregulation of Cyclin B1 by miRNA and its implications in cancer. Nucleic Acids Res..

[15] Raghavan P.C., Jeong K.J., Pradeep S., Silva A.M., Yu S.X., Liu W.B., Moss T., Aguayo C.R., Zhang D., Ram P. (2016). Direct upregulation of STAT3 by microRNA-551b-3p deregulates growth and metastasis of ovarian cancer. Cell Rep..

[16] Mogilyansky E., Rigoutsos I. (2013). The miR-17/92 cluster: A comprehensive update on its genomics, genetics, functions and increasingly important and numerous roles in health and disease. Cell Death Differ..

[17] Poitz D.M., Augstein A., Gradehand C., Ende G., Schmeisser A., Strasser R.H. (2013). Regulation of the Hif-system by micro-RNA 17 and 20a-role during monocyte-to-macrophage differentiation. Mol. Immunol..

[18] Cai Y.H., Chen H., Mo X.W., Tang Y.Y., Xu X.C., Zhang A.M., Lun Z.R., Lu F.L., Wang Y., Shen J.L. (2014). Toxoplasma gondii inhibits apoptosis via a novel STAT3-miR-17-92-Bim pathway in macrophages. Cell. Signal..

[19] Williams A., Dougal D.M., Jenkins W., Greene N., DeVane C.W., Kimbro K.S. (2019). Serum miR-17 levels are downregulated in obese, African American women with elevated HbA1c. J. Diabetes Metab. Disord..

[20] Zhang S.N., Wang L., Wang L., Chen Y.R., Li F.G. (2019). miR-17-5p affects porcine granulosa cell growth and oestradiol synthesis by targeting E2F1 gene. Reprod. Domest. Anim..

[21] Cao Y., Yang Q., Mai Q.Q., Wuliu J.X., Deng K.X. (2024). Relationship between triglyceride-glucose index and endometriosis: A cross-sectional analysis. BMC Womens Health.

[22] Zhang C.H., Liu X.Y., Wang J. (2023). Essential Role of Granulosa Cell Glucose and Lipid Metabolism on Oocytes and the Potential Metabolic Imbalance in Polycystic Ovary Syndrome. Int. J. Mol. Sci..

[23] Pan Q.W., Wang Y., Liu J.H., Jin X.J., Xiang Z., Li S.Q., Shi Y.M., Chen Y.F., Zhong W.T., Ma X.T. (2023). MiR-17-5p Mediates the Effects of ACE2-Enriched Endothelial Progenitor Cell-Derived Exosomes on Ameliorating Cerebral Ischemic Injury in Aged Mice. Mol. Neurobiol..

[24] Shi Y.P., Liu G.L., Li S., Liu X.L. (2020). miR-17-5p knockdown inhibits proliferation, autophagy and promotes apoptosis in thyroid cancer via targeting PTEN. Neoplasma.

[25] Liu H.T., Luo C.P., Jiang M.J., Deng Z.J., Teng Y.X., Su J.Y., Pan L.X., Ma L., Guo P.P., Zhong J.H. (2023). miR-17-5p slows progression of hepatocellular carcinoma by downregulating TGFβR2. Clin. Transl. Oncol..

[26] Cox J.F., Navarrete F., Carrasco A., Dorado J., Saravia F. (2019). Effect of bST administration on plasma concentrations of IGF-I and follicular dynamics and ovulation during the interovulatory cycle of sheep and goats. Theriogenology.

[27] Maalouf S.W., Liu W.S., Pate J.L. (2016). MicroRNA in ovarian function. Cell Tissue Res..

[28] Donadeu F.X., Sanchez J.M., Mohammed B.T., Ioannidis J., Stenhouse C., Maioli M.A., Esteves C.L., Lonergan P. (2020). Relationships between size, steroidogenesis and miRNA expression of the bovine corpus luteum. Theriogenology.

[29] Wang X.F., Yang J.H., Li H.Y., Mu H.B., Zeng L., Cai S.Y., Su P., Li H.B., Zhang L., Xiang W.P. (2023). miR-484 mediates oxidative stress-induced ovarian dysfunction and promotes granulosa cell apoptosis via SESN2 downregulation. Redox Biol..

[30] Ma R.J., Zhang M., Wu J.S., Wang Z.P., Wang G.L., He N., Luo M.J., Tan J.H. (2024). Role of miRNAs in glucose metabolism of mouse cumulus cells. Biol. Reprod..

[31] Cui X.R., Wang H.H., Wu X.Q., Huo K., Jing X. (2021). Increased expression of KPNA2 predicts unfavorable prognosis in ovarian cancer patients, possibly by targeting KIF4A signaling. J. Ovarian Res..

[32] Wang W.J., Miyamoto Y.C., Chen B.B., Shi J.Z., Diao F.Y., Zheng W., Li Q., Yu L., Li L., Xu Y. (2023). Karyopherin α deficiency contributes to human preimplantation embryo arrest. J. Clin. Investig..

[33] Madhumita M., Paul S. (2022). A review on methods for predicting miRNA-mRNA regulatory modules. J. Integr. Bioinform..

[34] Yao X.L., El-Samahy M.A., Li X.D., Bao Y.J., Guo J.H., Yang F., Wang Z.B., Li K., Zhang Y.L., Wang F. (2022). LncRNA-412.25 activates the LIF/STAT3 signaling pathway in ovarian granulosa cells of Hu sheep by sponging miR-346. FASEB J..

[35] Gui H.B., Li F., Chen C., Zhu Q.Y., Zhang C.J., Zhang J., Meng C.H., Qian Y., Cao S.X., Li Y.X. (2023). miR-27a-3p targets NR5A2 to regulate CYP19A1 expression and 17-β estradiol synthesis in ovine granulosa cells. Anim. Reprod. Sci..

[36] Li L.C., Okino S.T., Zhao H., Pookot D., Place R.F., Urakami S.J., Enokida H., Dahiya R. (2006). Small dsRNAs induce transcriptional activation in human cells. Proc. Natl. Acad. Sci. USA.

[37] Campbell B.K., Onions V., Kendall N.R., Guo L., Scaramuzzi R.J. (2010). The effect of monosaccharide sugars and pyruvate on the differentiation and metabolism of sheep granulosa cells In Vitro. Reproduction.

[38] Somchit A., Campbell B., Khalid M., Kendall N., Scaramuzzi R. (2007). The effect of short-term nutritional supplementation of ewes with lupin grain (*Lupinus luteus*), during the luteal phase of the estrous cycle on the number of ovarian follicles and the concentrations of hormones and glucose in plasma and follicular fluid. Theriogenology.

[39] Duan T.F., Li L., Tan Y., Li Y.Y., Pang B.P. (2021). Identification and functional analysis of microRNAs in the regulation of summer diapause in *Galeruca daurica*. Comp. Biochem. Physiol. Part D Genom. Proteom..

[40] Langmead B. (2010). Aligning short sequencing reads with Bowtie. Curr. Protoc. Bioinform..

[41] Kozomara A., Griffiths-Jones S. (2010). miRBase: Integrating microRNA annotation and deep-sequencing data. Nucleic Acids Res..

[42] Love M.I., Huber W.G., Anders S. (2014). Moderated estimation of fold change and dispersion for RNA-seq data with DESeq2. Genome Biol..

[43] Betel D., Wilson M., Gabow A., Marks D.S., Sander C. (2008). The microRNA.org resource: Targets and expression. Nucleic Acids Res..

[44] Agarwal V., Bell G.W., Nam J.W., Bartel D.P. (2015). Predicting effective microRNA target sites in mammalian mRNAs. Elife.

