# Supplementary material for: miR-17-5p-Mediated RNA Activation Upregulates KPNA2 Expression and Inhibits High-Glucose-Induced Apoptosis of Sheep Granulosa Cells
Source: Int J Mol Sci. 2025 Jan 23;26(3):943. doi: 10.3390/ijms26030943 (PMC11817598; doi:10.3390/ijms26030943)
Supplement: Supplementary file 1 [file ijms-26-00943-s001.zip › Figure S2.pdf]

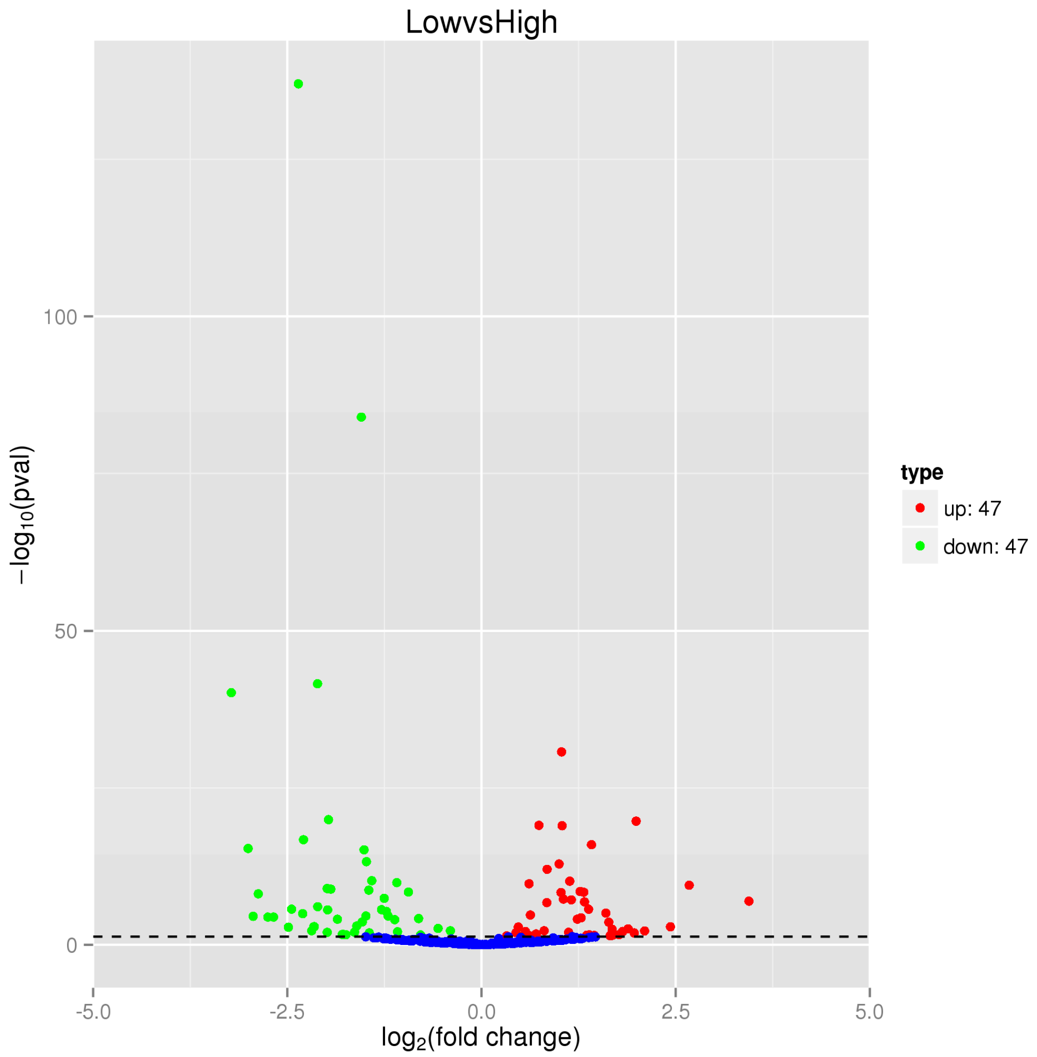

Figure S2: Volcano diagram of differentially expressed miRNA in granulosa cells between high and low glucose group; up− and down regulated genes are colored in red and green, respectively.
